# Next-Generation Multitarget Stool DNA vs Fecal Immunochemical Test in Colorectal Cancer Screening

**DOI:** 10.1001/jamainternmed.2024.6149

**Published:** 2024-11-18

**Authors:** Teresa Seum, Tobias Niedermaier, Thomas Heisser, Michael Hoffmeister, Hermann Brenner

**Affiliations:** 1 Division of Clinical Epidemiology and Aging Research, German Cancer Research Center (DKFZ), Heidelberg, Germany; 2 Heidelberg Medical Faculty, Heidelberg University, Heidelberg, Germany; 3 Department of Biometry and Bioinformatics, Institute for Medical Information Processing, Biometry, and Epidemiology, Ludwig Maximilian University of Munich, Munich, Germany; 4 German Cancer Consortium (DKTK), German Cancer Research Center (DKFZ), Heidelberg, Germany

## Abstract

This diagnostic study compares the specificity and sensitivity of comercial fecal immunochemical tests with a multitarget stool DNA test.

Fecal immunochemical tests (FITs) are the most widely used noninvasive colorectal cancer (CRC) screening tests.^1^ In 2014, a US/Canadian screening study reported higher sensitivity of a multitarget stool DNA test (MSDT), which combined fecal hemoglobin measurement with molecular DNA markers, compared with a commercial FIT. However, this increase in sensitivity was accompanied by a substantial loss of specificity.^2^ Despite 20-fold higher per-sample costs, use of MSDT-based screening strongly increased in the US in recent years.^3^ Recently, Imperiale et al^4^ evaluated diagnostic performance of a next-generation MSDT (NG-MSDT) in another screening colonoscopy cohort (BLUE-C study). Sensitivity was higher than that of a commercial FIT, although again specificity was lower. It was previously shown that lowering the positivity threshold of a commercial quantitative FIT could enhance diagnostic performance to be comparable to a multitarget stool ribonucleic acid (RNA) test,^5^ prompting an evaluation of whether the same approach could yield similar outcomes with the NG-MSDT.

## Methods

This study was approved by was approved by the Heidelberg Medical Faculty of Heidelberg University and participants gave written informed consent. Our analyses, which were performed in the Spring of 2024, are based on the BLITZ study, a large screening colonoscopy cohort from Germany.^5,6^ Among 10 061 participants recruited in 2008 to 2020, in whom the same commercial FIT (FOB Gold; Sentinel Diagnostics) was used, we made the following exclusions to make the study population as comparable as possible to that of the BLUE-C study: younger than 40 years, history of CRC or inflammatory bowel disease, colonoscopy fewer than 10 years before, inadequate bowel preparation, incomplete colonoscopy, type of polyp(s) unspecified in colonoscopy report, and stool sampling after colonoscopy.

We summarized and compared participant characteristics in the BLUE-C study and the BLITZ study by descriptive statistics. Using the BLITZ study sample, we derived FIT sensitivities for detecting the following findings: any CRC, CRC stages I to III, any advanced precancerous lesion, and any advanced neoplasm (CRC or advanced precancerous lesion). Specificity was derived as proportion of negative results in the absence of advanced neoplasms. To enhance comparability, diagnostic performance characteristics of the FIT were determined for the cutoff recommended by the manufacturer (17 μg hemoglobin per gram feces) and lower cutoffs yielding either the same specificity or the same sensitivity for detection of any advanced neoplasm as reported for the NG-MSDT.^4^ In both studies, colonoscopy served as the gold standard to determine stool test performance.

## Results

Overall, 7190 participants were finally included. Sex distribution and mean age were very similar in the BLITZ study (51.6% women, 61.3 years) and the BLUE-C study^4^ (53.2%, 63.0 years) (Table 1). However, the BLUE-C study had a higher proportion of participants aged 65 years or older due to intentional oversampling of older adults. Prevalence of CRC was slightly higher in BLITZ, but prevalence of advanced precancerous lesions was similar in both studies.

**Table 1.** Main Characteristics of the Study Population in the BLUE-C and the BLITZ Study Population

Characteristic	Study, No. (%)	
	BLUE-C^a^	BLITZ
Total No.	20 176	7190
Country/years	United States, 2019-2023	Germany, 2008-2020
Sex		
Men	9436 (46.8)	3478 (48.4)
Women	10 740 (53.2)	3712 (51.6)
Age, y		
Mean (SD) [range]	63.0 (7.3) [≥40]	61.3 (7.2) [≥40]
<50	377 (1.9)	168 (2.3)
50-64	10 222 (50.7)	4763 (66.2)
65-75	8805 (43.6)	1999 (27.8)
≥76	772 (3.8)	260 (3.6)
Most advanced finding at colonoscopy		
CRC	98 (0.5)	57 (0.8)
Thereof CRC, stage I-III	82 (0.4)	48 (0.7)
Advanced precancerous lesion^b^	2144 (10.6)	754 (10.5)
Any advanced neoplasia^c^	2242 (11.1)	811 (11.3)
No advanced neoplasia	17 934 (88.9)	6379 (88.7)

Abbreviation: CRC, colorectal cancer.

^a^ Data extracted from Imperiale et al.^4^

^b^ Defined as adenomas or sessile serrated polyps ≥1 cm, lesions with villous histologic features, or high-grade dysplasia.

^c^ Includes CRC or advanced precancerous lesions.

When evaluated at the cutoff recommended by the manufacturer (17 μg/g), sensitivities of the FIT in BLITZ were consistently lower, and specificity was higher than reported for the NG-MSDT in BLUE-C (Table 2). However, after lowering the cutoff of the FIT to 11.7 μg/g to yield comparable specificity (90.6%), even slightly higher sensitivity for CRC (94.7% vs 93.9%) was observed, along with slightly lower sensitivity for advanced precancerous lesions (38.3% vs 43.4%). By further lowering the FIT cutoff to 10 μg/g to yield the same sensitivity for any advanced neoplasia (45.4%), a 96.5% sensitivity for CRC was reached, at only modestly lower specificity (89.0%) than reported for the NG-MSDT.

**Table 2.** Comparison of Sensitivity and Specificity for Detecting CRC or Advanced Precancerous Lesions Reported for the NG-MSDT and the FIT in the BLUE-C Study^4^ and in the BLITZ Study

Metric	Outcome	Study				
		BLUE-C^a^		BLITZ		
		NG-MSDT	FIT (cutoff 20 μg/g)^b^	FIT^c^ (cutoff 17 μg/g)^d^	FIT^c^ (cutoff 11.7 μg/g)^e^	FIT^c^ (cutoff 10 μg/g)^f^
Sensitivity (95% CI)	CRC, any stage	93.9 (87.1-97.7)	67.3 (57.1-76.5)	89.5 (78.5-96.0)	94.7 (85.4-98.9)	96.5 (87.9-99.6)
	CRC, stage I-III	92.7 (84.8-97.3)	64.6 (53.3-74.9)	87.5 (74.8-95.3)	93.8 (82.8-98.7)	95.8 (85.7-99.5)
	Advanced precancerous lesion	43.4 (41.3-45.6)	23.3 (21.5-25.2)	32.6 (29.3-36.1)	38.3 (34.8-41.9)	41.5 (38.0-45.1)
	Any advanced neoplasm	45.6 (43.6-47.7)	25.2 (23.5-27.1)	36.6 (33.3-40.0)	42.3 (38.9-45.8)	45.4 (41.9-48.9)
Specificity (95% CI)	No advanced neoplasm	90.6 (90.1-91.0)	94.8 (94.4-95.1)	93.2 (92.6-93.8)	90.6 (89.8-91.3)	89.0 (88.2-89.8)

Abbreviations: CRC, colorectal cancer; FIT, fecal immunochemical test; NG-MSDT, next-generation multitarget stool DNA test.

^a^ Results extracted or derived from Table 1 in the study by Imperiale et al.^4^

^b^ OC Auto FIT (Polymedco).

^c^ FOB Gold (Sentinel Diagnostics).

^d^ Cutoff recommended by the manufacturer.

^e^ Cutoff of 11.7 μg/g adjusted to yield comparable specificity as the MSDT for indicating absence of any advanced neoplasia.

^f^ Cutoff of 10 μg/g adjusted to yield comparable sensitivity as the MSDT for detecting presence of any advanced neoplasia.

## Discussion

A commercial FIT can achieve similar diagnostic results to the newer stool DNA test, as was shown before for the first generation of both multitarget stool DNA and multitarget stool RNA tests.^5^ Despite the limitation of being an indirect comparison, the findings of this study suggest that lowering the positivity threshold of a high-quality FIT may be a much more economical way to increase sensitivity of noninvasive CRC screening. Further research to enhance both sensitivity and specificity of noninvasive CRC screening tests is highly desirable.

## Notes

Supplement. Data Sharing Statement
